# Synaptic remodeling of neuronal circuits in early retinal degeneration

**DOI:** 10.3389/fncel.2015.00395

**Published:** 2015-10-07

**Authors:** Florentina Soto, Daniel Kerschensteiner

**Affiliations:** ^1^Department of Ophthalmology and Visual Sciences, Washington University School of Medicine in St. LouisSt. Louis, MO, USA; ^2^Department of Anatomy and Neurobiology, Washington University School of Medicine in St. LouisSt. Louis, MO, USA; ^3^Department of Biomedical Engineering, Washington University School of Medicine in St. LouisSt. Louis, MO, USA; ^4^Hope Center for Neurological Disorders, Washington University School of Medicine in St. LouisSt. Louis, MO, USA

**Keywords:** retina, circuit remodeling, lamination, mosaic, developmental plasticity

## Abstract

Photoreceptor degenerations are a major cause of blindness and among the most common forms of neurodegeneration in humans. Studies of mouse models revealed that synaptic dysfunction often precedes photoreceptor degeneration, and that abnormal synaptic input from photoreceptors to bipolar cells causes circuits in the inner retina to become hyperactive. Here, we provide a brief overview of frequently used mouse models of photoreceptor degenerations. We then discuss insights into circuit remodeling triggered by early synaptic dysfunction in the outer and hyperactivity in the inner retina. We discuss these insights in the context of other experimental manipulations of synaptic function and activity. Knowledge of the plasticity and early remodeling of retinal circuits will be critical for the design of successful vision rescue strategies.

## Mouse models of retinal degeneration

Photoreceptors are the most common target of neurodegenerative diseases in humans. Inherited forms of retinal degeneration—including retinitis pigmentosa, cone-rod dystrophies and Leber's congenital amaurosis—are a heterogeneous group of mostly monogenic diseases, for which mutations in more than 190 genes have been identified (Daiger et al., 2013). The genetic diversity of retinal degenerations is accompanied by variability in their clinical manifestations including in age of onset, degree and order of rod vs. cone death, involvement of other retinal neurons, and presence of additional neurological symptoms (Wright et al., 2010).

Spontaneous mutations or targeted deletions of genes associated with human retinal degeneration cause similar photoreceptor dysfunction and death in mouse retinae. One of the most widely used models of retinitis pigmentosa, the *rd1* mouse, harbors a spontaneous mutation in the phosphodiesterase 6β (*PDE6B*) gene, a common locus for mutations in retinitis pigmentosa (Chang et al., 2002). Retinal degeneration in *rd1* mice begins around postnatal day 8 (P8) with the death of rod photoreceptors (Carter-Dawson et al., 1978). Another spontaneous missense mutation of the *PDE6B* gene was identified in *rd10* mice, in which rods start to degenerate at P18 (Chang et al., 2002). Similar to retinitis pigmentosa, rod degeneration is followed by cone death in both *rd1* and *rd10* mice. The delayed onset of photoreceptor degeneration in *rd10* mice makes them a more accurate model of retinitis pigmentosa compared to *rd1* mice. Mice lacking the cone-rod homeobox gene (*Crx*^−∕−^ mice) have been proposed as a model for Leber's congenital amaurosis, as deletion mutations in Crx cause both dominant and recessive forms of Leber's congenital amaurosis (Veleri et al., 2015). In *Crx*^−∕−^ mice, photoreceptors fail to develop outer segments and thus never respond to light (Furukawa et al., 1999). In addition, the formation of synapses between photoreceptors and bipolar cells is disrupted (Furukawa et al., 1999). However, photoreceptor death is delayed in *Crx*^−∕−^ compared to *rd1* and *rd10* mice and does not overlap with retinal development (Pignatelli et al., 2004). There are several other mouse models of retinal degeneration caused by mutation or deletion of genes involved in phototransduction or photoreceptor synaptic function (Chang et al., 2005).

Together, these mouse models have proven useful in determining mechanisms and progression of photoreceptor degeneration and its consequences for downstream neural circuits. In addition, they are invaluable tools for developing strategies to halt degeneration and for testing novel approaches to restore vision.

## Spontaneous retinal activity

Spontaneous activity propagates through many parts of the developing nervous system and regulates synaptic refinement of emerging circuits (Kerschensteiner, 2013). In the visual system, waves of spontaneous activity generated in the inner retina propagate through subcortical visual areas and dictate activity up to primary visual cortex (Meister et al., 1991; Ackman et al., 2012). Retinal waves influence circuit development in the retina, promote eye-specific segregation and topographic refinement of retinofugal projections, and adjust geniculo-cortical and cortico-collicular connectivity (Kerschensteiner, 2013; Kirkby et al., 2013; Ackman and Crair, 2014). Across many species, retinal waves mature in three stereotypic stages, in which different circuit mechanisms give rise to activity patterns with distinct spatiotemporal properties (Wong, 1999; Blankenship and Feller, 2010). Retinal waves subside around the time of eye opening (~P15 in mice) as photoreceptor input in the outer retina begins to drive bipolar cells (Demas et al., 2003). The transition from waves to vision is unperturbed by dark rearing, indicating that light-evoked signals from photoreceptors are not required for this process (Demas et al., 2003).

Retinal waves are preserved in mouse models of inherited retinal degenerations. However, at the time when patterned spontaneous activity normally subsides, ganglion cells in the respective retinae begin to exhibit oscillatory hyperactivity (Margolis et al., 2008; Stasheff, 2008; Borowska et al., 2011; Soto et al., 2012; Yee et al., 2012). Spontaneous hyperactivity co-exists with remnant light responses in *rd10* mice (Stasheff et al., 2011). Moreover, similar hyperactivity is observed in null mutants of nyctalopin (*nob* mice) (Demas et al., 2006), which mimic congenital stationary night blindness, a heterogeneous group of diseases in which signaling from photoreceptors to ON bipolar cells—including rod bipolar cells—but not OFF bipolar cells is disrupted (McCall and Gregg, 2008). In *nob* mice and congenital stationary night blindness patients, photoreceptors do not degenerate (Gregg et al., 2007). Spontaneous oscillatory hyperactivity thus seems to be a common and early feature of diseases involving disrupted synaptic communication between photoreceptors and bipolar cells. The circuit mechanisms underlying spontaneous hyperactivity are under investigation. Results so far suggest that oscillations arise presynaptic to retinal ganglion cells in electrically coupled networks of ON cone bipolar and AII amacrine cells (Margolis et al., 2008, 2014; Borowska et al., 2011; Trenholm et al., 2012; Choi et al., 2014).

Given the early onset of spontaneous hyperactivity, recent studies have explored to what extent inherited retinal degenerations interfere with normal development of circuits in the inner retina. Here, we review findings from these studies in the context of other experimental activity manipulations. In addition, we discuss insights into early changes in the outer retina that accompany photoreceptor dysfunction and/or degeneration. We divide our discussion into three parts, dealing separately with critical steps along the way to precise retinal circuits.

## Retinal lamination

**Figure 1:**
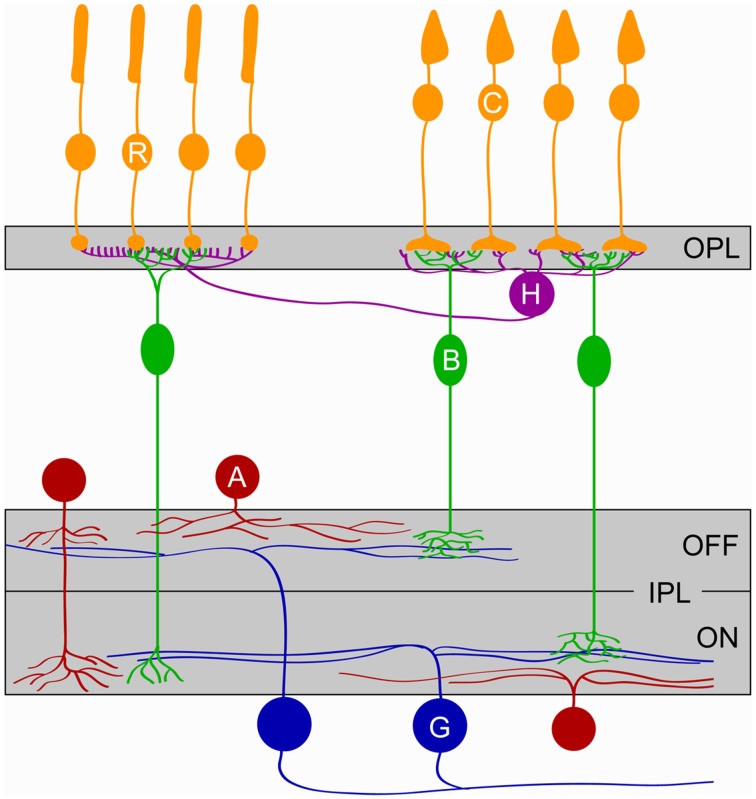
**Schematic of the retina**. The five major classes of retinal cells and their lamination are depicted. Cones (C, *orange*), Rods (R, *orange*), Horizontal cells (H, *purple*), Bipolar cells (B, *green*), Amacrine cells (A, *red*), and Ganglion cells (G, *blue*). OPL, outer plexiform layer; IPL, inner plexiform layer; ON and OFF denote the parts of the IPL where circuits responding to the onset and the offset of light, respectively, stratify.

The retina consists of more than 60 different types of neurons belonging to five cell classes, arranged in three somatic layers separated by two synaptic (or plexiform) layers (Figure 1) (Masland, 2012). Photoreceptors, horizontal and bipolar cells target neurites to the outer plexiform layer (OPL) where they form characteristic tripartite synapses. In mice, connections in the OPL are functional by P14 (Blanks et al., 1974), allowing the transfer of visual information from the time of eye opening. Bipolar cells carry photoreceptor signals from the OPL to the inner plexiform layer (IPL) where their axons establish synapses with amacrine cells and ganglion cells, the output neurons of the eye. The IPL can be divided into five anatomically distinct sublaminae (S1-S5). Neurons activated by light decrements (OFF) stratify in the outer two sublaminae (S1-S2), whereas neurons activated by light increments (ON) stratify in the inner three (S3-S5). Cell-type-specific lamination patterns of neurites at precise depths of the IPL restrict potential connectivity and thus contribute to the synaptic specificity of retinal circuits (Sanes and Zipursky, 2010; Masland, 2012).

In mouse models of retinal degeneration, photoreceptors, horizontal cells, and bipolar cells stratify normally in the OPL by P15. However, by 1 month of age, horizontal cell axons begin to extend aberrant processes from the OPL into the surrounding nuclear layers of *rd1* and *rd10* mouse retinae (Phillips et al., 2010). Disruption of horizontal cell stratification has been observed in mice in which genes critical for transmitter release from photoreceptors such as the ribbon anchoring protein bassoon (Specht et al., 2007), the calcium binding protein 4 (Haeseleer et al., 2004), the calcium channel subunits α1F (Chang et al., 2006), and α2/δ4 (Wycisk et al., 2006), or genes involved in synapse formation (Soto et al., 2013) were deleted. Together these findings suggest that normal synaptic input from photoreceptors maintains stratification of horizontal cell neurites and that the sprouting of ectopic horizontal cell processes in models of retinal degeneration is the result of synaptic dysfunction. By contrast, photoreceptors appear to promote maintenance of bipolar cell dendrites independent of synaptic activity as bipolar cell dendrites are preserved in *Crx*^−∕−^ mice but retract from the OPL of *rd1* mice by 3 weeks of age (Gargini et al., 2007).

Aberrant spontaneous activity in mouse models of retinal degenerations does not appear to alter neurite stratification in the IPL. Thus, cell-type-specific lamination patterns of rod, and ON and OFF cone bipolar cells are preserved in *Crx*^−∕−^ mice. Moreover, the neurites of starburst and VGluT3-expressing amacrine cells and dendrites of ON and OFF ganglion cells target the correct IPL sublaminae in *Crx*^−∕−^ retinae (Soto et al., 2012). Similarly, laminar targeting of rod and cone bipolar cell axons and stratification of starburst, dopaminergic and AII amacrine cell arbors are unchanged in *rd1* mice (Pignatelli et al., 2004). *Nob* mice inner retinae are also normal (Pardue et al., 1998). Complementing these observations, ON bipolar cell axons and ON ganglion cell dendrites were found to co-stratify correctly in mice in which glutamate release from ON bipolar cells is inhibited by transgenic expression of tetanus toxin (Kerschensteiner et al., 2009). However, other studies have suggested that pruning of incorrectly placed retinal ganglion cell dendrites may be altered in dark-reared mice (Tian and Copenhagen, 2003; Xu and Tian, 2007) and cats in which the ON pathway is pharmacologically suppressed (Bodnarenko et al., 1995). As the timing and strategies of stratification differ between retinal ganglion cell types (Mumm et al., 2006; Kim et al., 2010), the influence of activity may vary between cell types as well. Nonetheless, for amacrine and bipolar cells and at least a subset of retinal ganglion cell types activity plays a minor role in this process, which instead seems to be governed by cell adhesion and repulsive guidance molecules (Fuerst et al., 2008; Yamagata and Sanes, 2008; Matsuoka et al., 2011).

## Retinal mosaics and neurite coverage

In addition to the vertical organization of neurites into layers, the cell bodies of retinal neurons show regular horizontal distributions called mosaics (Wässle, 2004). This arrangement helps neuronal cell types cover the retina evenly and represent visual space homogeneously. In *rd1* mice, the spatial organization and regularity of horizontal cells is preserved up to at least 1 month of age (Rossi et al., 2003) despite almost complete photoreceptor degeneration by this time. Similarly, the mosaic distribution and dendritic arbor size of (type 7) cone bipolar cells is unchanged in 3 weeks old *rd1* mice (Chen et al., 2012). Moreover, the number and distribution of horizontal and rod bipolar cells is maintained until at least 1.5 months of age in *rd10* retinae (Gargini et al., 2007). Likewise, dark rearing does not affect the number of horizontal cells up to at least 2 months of age (Raven et al., 2008).

In the inner retina of *Crx*^−∕−^ mice, the density of posterior-motion-preferring direction selective ganglion cells and the regularity of their distribution were unchanged compared to wild-type litter mates. The same result was obtained when the distribution of ON and OFF starburst amacrine cells was analyzed (Soto et al., 2012). In addition, the number of cells and mosaics of starburst, dopaminergic and AII amacrine cells were unchanged in 3 months old *rd1* mice (Strettoi et al., 2002, 2003). Together these observations suggest that neurons in the outer and inner retina establish and maintain mosaic distributions independent of photoreceptor input and spontaneous activity patterns.

Within each mosaic, dendrites of neighboring neurons overlap by a cell-type-specific amount. In addition to contributing to the size of receptive fields, lateral arbor growth thus determines the retinal coverage of the respective cell type. Contact-mediated lateral interactions between retinal neurons of the same type have been shown to regulate the size and overlap of their dendrites (Perry and Linden, 1982; Fuerst et al., 2008, 2009; Huckfeldt et al., 2009). Dendritic field sizes of horizontal (Raven et al., 2008) and bipolar cells are indistinguishable between retinae with widely different photoreceptor densities, consistent with a dominant role for homotypic interactions. By contrast, changes in the complexity of horizontal cell dendrites were observed in retinae of cone-less and of *Cacna1f*^−∕−^ mice early in development (P10) (Raven et al., 2008), indicating that input from photoreceptors might regulate branching within the homotypically defined arbor territories. In the inner retina, studies in *rd1, rd10*, and *Crx*^−∕−^ mice showed retinal ganglion cells retaining their morphology up to at least 6 months of age (Mazzoni et al., 2008; Soto et al., 2012; Lin and Peng, 2013) (but see Damiani et al., 2012). Similarly, suppression of glutamate release from ON bipolar cells does not affect the arbor size or branch complexity of retinal ganglion cells dendrites (Kerschensteiner et al., 2009), arguing that activity plays a minor role in their development and maintenance.

## Synaptic changes in models of retinal degeneration

Downregulation and mislocalization of proteins of the pre- and post-synaptic machinery in the OPL precedes photoreceptor degeneration in *rd1* (Strettoi et al., 2002), *rd10* (Puthussery and Taylor, 2010), and *Crx*^−∕−^ (Pignatelli et al., 2004; Soto et al., 2013) mice. In addition, electron microscopic analysis of *Crx*^−∕−^ retinae revealed developmental ultrastructural abnormalities of photoreceptor synapses (Morrow et al., 2005). Dark rearing was recently shown to affect localization of mGluR6 receptors to synapses of ON cone bipolar cells (Dunn et al., 2013), and functional deficits in glutamate receptor signaling in bipolar cell dendrites have been observed prior to degeneration in *rd1* and *rd10* mice (Puthussery et al., 2009). In contrast, an increase in the expression of glutamate receptor 2 subunits in the OPL was detected in light-induced retinal degeneration in adult mice, but levels returned to normal 30 days after the insult (Lin et al., 2012). Together these studies suggest that synaptic changes are a common early event in retinal degenerations and that abnormal light-evoked activity may contribute to the dysfunction of connections in the OPL.

Rod bipolar cell dendrites avoid cones in normal retinae, but form synapses with cones in *rd1* and rhodopsin KO (*Rho*^−∕−^) retinae, in the absence of rods (Peng et al., 2000; Haq et al., 2014). Conversely, cone bipolar cells form ectopic synapses with rods in cyclic nucleotide gated channel α3 KO mice (*CNGA3*^−∕−^), in which cones do not respond to light (Haverkamp et al., 2006). Interestingly, no ectopic synapses between bipolar cells and photoreceptors are observed when both rods and cones are nonfunctional, indicating that these connections represent an effort of bipolar cells to restore input activity (Haverkamp et al., 2006). Rod bipolar and horizontal cells extend dendritic arbors into the outer nuclear layer to form ectopic synapses with retracting rod terminals in mice deficient for the photoreceptor calcium binding protein 4 (Haeseleer et al., 2004), the calcium channel subunit α1F (Chang et al., 2006) or Bassoon (Specht et al., 2007, 3–4 weeks mouse). In these animals, unlike *rd1* or *Rho*^−∕−^, some rod function remains which appears sufficient to sustain the formation of ectopic synapses.

**Figure 2:**
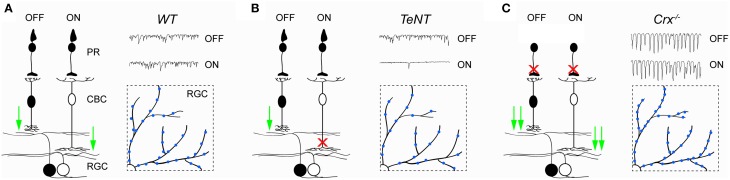
**Activity-dependent remodeling of circuits in the inner retina**. **(A–C)** Illustrate synaptic function and BC-RGC connectivity in wild-type (*WT*, **A**) retinae, retinae in which ON bipolar cells express tetanus toxin (*TeNT*, **B**), and retinae of *Crx*^−∕−^ mice (*Crx*^−∕−^, **C**). In *TeNT* retinae, release of glutamate from OFF bipolar cells is unaffected, whereas release of glutamate from ON bipolar cells onto ganglion cells is suppressed. In contrast, release of glutamate from both ON and OFF cone bipolar cells onto ganglion cells is increased in *Crx*^−∕−^ mice. Insets represent partial top-down views of ganglion cell dendrites and their excitatory synapses (*blue*). The number of excitatory synapses increases in *Crx*^−∕−^ and decreases in *TeNT* mice compared to *WT* retinae. PR, photoreceptor; CBC, cone bipolar cell; RGC, retinal ganglion cell.

Loss of photoreceptor input produces oscillatory hyperactivity in the inner retina, elevating glutamate release from bipolar cells (Menzler and Zeck, 2011; Trenholm and Awatramani, 2015). In *Crx*^−∕−^ mice, this increase in activity was shown to enhance the formation of excitatory synapses on ganglion cells dendrites (Figure 2) (Soto et al., 2012). Differences in synaptogenesis between *wild-type* and *Crx*^−∕−^ mice emerge around P15 and stabilize by P25, even though activity levels continue to increase in *Crx*^−∕−^ mice up to at least 6 month of age (Soto et al., 2012). Similarly, increased levels of excitatory glutamate receptor 1, GABA receptor α1 and glycine receptor α1/α2 subunits were found in the IPL of 2–3 month old *rd1* retinas (Srivastava et al., 2015). Interestingly, a decrease in the formation of excitatory synapses was observed in retinae in which glutamate release from ON bipolar cells is inhibited by transgenic expression of tetanus toxin (Figure 2) (Kerschensteiner et al., 2009). This indicates that synaptogenesis is regulated by input activity in a bidirectional manner. Moreover, these studies suggest that in mice the critical period for synaptic refinement in the inner retina extends approximately from the end of the first to the fourth week of postnatal life, when the majority of connections are established (Kerschensteiner et al., 2009; Morgan et al., 2011).

## Outlook

Animal models help elucidate the pathogenesis of retinal degenerative diseases in humans and aid in the development of strategies to restore vision. The initial steps in retinal degenerations involve abnormal synaptic transmission between photoreceptors and bipolar cells in the outer retina. These early changes in the OPL affect not only the development of dendritic arbors of horizontal and bipolar cells but also result in rhythmic hyperactivity in the inner retina. Changes in inner retinal activity in turn affect synapse formation between bipolar and ganglion cells during critical periods of development. Future studies are needed to determine if this plasticity can be elicited outside of early development and if it occurs in human patients with retinal degenerative diseases. In addition, it remains to be investigated how plasticity can be reactivated to optimally integrate newly generated signals (e.g., optogenetics; Busskamp et al., 2012) and neurons (e.g., generated from stem cells; Wiley et al., 2015) into circuits in vision restoration strategies.

### Conflict of interest statement

The authors declare that the research was conducted in the absence of any commercial or financial relationships that could be construed as a potential conflict of interest.
